# A Framework for Evaluating Local Adaptive Capacity to Health Impacts of Climate Change: Use of Kenya’s County-Level Integrated Development Plans

**DOI:** 10.5334/aogh.4266

**Published:** 2024-02-14

**Authors:** Megan Kowalcyk, Samuel Dorevitch

**Affiliations:** 1 Division of Environmental and Occupational Health Sciences, University of Illinois Chicago, School of Public Health, Chicago IL, US

**Keywords:** Health impacts of climate change, adaptation planning, adaptive capacity, health sector adaptation

## Abstract

**Background:**

Health National Adaptation Plans were developed to increase the capacity of low- and middle-income countries (LMICs) to adapt to the impacts of climate change on the health sector. Climate and its health impacts vary locally, yet frameworks for evaluating the adaptive capacity of health systems on the subnational scale are lacking. In Kenya, counties prepare county integrated development plans (CIDPs), which contain information that might support evaluations of the extent to which counties are planning climate change adaptation for health.

**Objectives:**

To develop and apply a framework for evaluating CIDPs to assess the extent to which Kenya’s counties are addressing the health sector’s adaptive capacity to climate change.

**Methods:**

CIDPs were analyzed based on the extent to which they addressed climate change in their description of county health status, whether health is noted in their descriptions of climate change, and whether they mention plans for developing climate and health programs. Based on these and other data points, composite climate and health adaptation (CHA) scores were calculated. Associations between CHA scores and poverty rates were analyzed.

**Findings:**

CHA scores varied widely and were not associated with county-level poverty. Nearly all CIDPs noted climate change, approximately half mentioned health in the context of climate change and only 16 (34%) noted one or more specific climate-sensitive health conditions. Twelve (25%) had plans for a sub-program in both adaptive capacity and environmental health. Among the 24 counties with plans to develop climate-related programs in health programs, all specified capacity building, and 20% specified integrating health into disaster risk reduction.

**Conclusion:**

Analyses of county planning documents provide insights into the extent to which the impacts of climate change on health are being addressed at the subnational level in Kenya. This approach may support governments elsewhere in evaluating climate change adaptation for health by subnational governments.

## Background

### Adaptive capacity, climate change, and health

Climate change is a threat to global health due to increasing exposure to climate-sensitive health hazards: heat, drought, flooding, sea-level rise, and the distribution of vector-borne diseases. Changes in the burden of disease due to these health hazards depend on both the adaptive capacity and sensitivity of a community [1]. Adaptive capacity is the ability of a system, such as the healthcare system, to reduce the adverse impacts of a stressor—such as climate change—on a system [1]. Sensitivity is the degree to which a system is affected by climate change, or susceptible to harm [1]. A function of sensitivity and adaptive capacity is vulnerability, or predisposition to be adversely impacted [1]. Consider two communities, both facing the same climate hazards and having comparable sensitivity to those hazards. The community with health care and public health systems that can withstand the impacts of the climate hazard, overall, will have lower vulnerability to the health effects of climate change. We refer to these as “health systems,” which are the network of hospitals, public health facilities, emergency response systems, outpatient care facilities, and pharmacies. While healthcare systems in high-income countries may have resources to rebuild following a climate disaster, healthcare systems in low- and middle-income countries (LMIC) may not. Increasing the adaptive capacity of healthcare systems in those countries can be accomplished through actions such as strengthening primary care services (to keep patients well), developing early warning systems for disasters, establishing multisectoral collaboration, educating the health workforce about climate-sensitive health conditions, and building climate resilient infrastructure, such as electrical grids, water infrastructure, and health care facilities [2].

### International adaptation plans

A framework for climate change adaptation was developed by the United Nations Framework Convention on Climate Change (UNFCCC) in 2011 [3,4]. That framework, the National Adaptation Plan (NAP), has two main objectives: to reduce vulnerability to climate change at the national level and to facilitate the integration of climate change adaptation into new and existing policies in LMIC [4]. The initial NAP guidance did not emphasize the health impacts of climate change or health sector adaptation in the context of climate change [4]. In 2014 the World Health Organization (WHO) filled this gap by developing guidelines for Health National Adaptation Plans (HNAPs) [5]. HNAPs consider the physical, social, and biological determinants of health [6]. The objectives of an HNAP are to reduce vulnerability, build adaptive capacity and resilience, and to facilitate integration of climate change adaptation into new and existing policies in LMIC [7]. HNAP guidance is intended to ensure that health risks of climate change are integrated into the overall NAP [5]. HNAPs should also ensure that climate-sensitive health outcomes are addressed and that the health sector can access adaptation funds. HNAPs should integrate health adaptation to climate change into national health systems [5,8]. Though NAPs have been submitted by 19 countries, only 4 countries have submitted HNAPs to the WHO: Ethiopia, Brazil, Fiji, and Kiribati [8]. In 2021, the WHO evaluated 19 NAPs submitted to UNFCCC to examine the extent to which health was considered in climate change adaptation [8]. That evaluation framework found that all 19 NAPs identified the health sector as being vulnerable to climate change. Importantly, the WHO evaluation noted that: “The conduct of health vulnerability assessments and the use of findings could be strengthened in many NAPs, such as through using context-specific local data, establishing baselines and projections, using a clear methodology, and establishing a clear link between the vulnerability assessment findings and proposed adaptation actions.”

Prior to the WHO’s development of HNAP’s, the WHO Regional Committee for Africa adopted the Adaptation to Climate Change in Africa Plan of Action for the Health Sector 2012–2016 (ACCAPAHS) [9]. The objectives of ACCAPAHS are to identify country-specific climate-sensitive health risks in Africa, strengthen national health systems, facilitate implementation of public health and environmental interventions, facilitate research on local health adaptation, and to facilitate implementation of adaptation strategies in other relevant sectors [9].

These adaptation frameworks—NAPs, HNAPs, and ACCAPAHS—call for a comprehensive assessment of vulnerability and adaptive capacity to climate change while considering the disproportionate burden of climate-sensitive health outcomes on vulnerable populations [7]. However, how the adaptive capacity of LMIC health systems should be assessed has not been specified. One of the aims of this research is to address that knowledge gap using information that has already been compiled by government agencies.

### Climate change policy in Kenya

Kenya is a lower-middle-income nation in Eastern sub-Saharan Africa with a population of 52.6 million people [10]. Kenya is experiencing the effects of climate change nationwide, including rising temperature, sea level rise, increased rainfall and floods in some areas, and droughts in others [11,12,13,14,15]. These changes in climate can lead to increases in malnutrition, vector-borne diseases (such as malaria and Rift Valley Fever), and waterborne diseases in the near to long-term future [16]. As of 2010, approximately 30% of children under 5 years old were stunted in Kenya, this is expected to increase and with increases in frequency and duration of droughts [17]. Increased precipitation and flooding are associated with malaria, Rift Valley fever, and increased incidence of cholera and diarrheal disease [18].

In its most recent NAP, the Kenyan government addressed health in the context of climate change, including proposed short- and medium-term actions to address health [11]. Those include the development of climate change and health vulnerability assessments, increasing public awareness of the connection between climate change and health, the need for climate change-related interventions for the health sector, and beginning or enhancing surveillance of climate change related diseases [11]. Other Kenyan policies address health, such as Health Policy 2017, Kenya Health Policy 2014–2030, Kenya Community Health Strategy 2020–2025, Kenya Health Sector Strategic Plan 2018–2023, and Universal Health Coverage plans as presented as a part of the Big Four Agenda in 2018 [11,19,20,21,24,25]. Health sector adaptation plans for climate change include recruitment of more technical staff, construction of mobile clinics, health education campaigns and enhanced surveillance [22]. These recent—but separate—policies for health (the Big Four Agenda) and climate (Climate Change Act of 2016) on a national scale attest to the focus the Kenyan government has placed on climate change and health in recent years [13,14,15,16,17]. However, there is very limited overlap in health policies and climate policies, which would be important for increasing adaptive capacity to the health impacts of climate change in Kenya.

### Kenya: Subnational vulnerability to climate change

Kenya has several distinct climate zones, including coastal areas, arid lands, tropical areas, and highlands, which present different health hazards [10]. For example, Mombasa, a city of 700,000 people on Kenya’s Indian Ocean coastline, is very vulnerable to sea level rise, where an estimated 17% of the city will be submerged when the sea level rises 0.3 m [23]. While sea level rise is a hazard of concern on the coast, floods and droughts are the hazards of greatest concern nationally [10]. Arid and semi-arid lands (ASAL), accounting for 88% of the land in Kenya, are the most vulnerable regions to the most adverse impacts of droughts [13]. Additionally, in ASAL regions of Kenya, precipitation level has a significant effect on child stunting with households that rely on surface water having a higher incidence of stunting [17,27,28]. Given the substantial variability within Kenya of climate, climate-sensitive health conditions, and social factors, it is important to know the extent to which county-level planning addresses the preparedness of the health sector for climate change.

### County integrated development plans

Following the passage of the Public Finance Management Act in 2012, every County Government in Kenya is required to develop a 5-year county integrated development plan (CIDP) [24]. CIDPs are intended to inform the county’s budget, sectoral, spatial, city, and municipal plans and reflect the midterm priorities of the county government [24]. CIDPs contain objectives, implementation plans, monitoring and evaluation plans, and reporting mechanisms. Following the initial CIDP for 2013–2017, all 47 counties have completed their CIDPs for the 2018–2022 period [24]. Given that climate change, health impacts, and sociodemographic characteristics vary at a subnational scale in Kenya, CIDPs provide an opportunity to evaluate the extent to which county officials address health in their preparations for climate change. The short- to medium-term goals as well as budgets spelled out in CIDPs are an opportunity to assess the extent to which climate change and health are being addressed jointly [25]. Additionally, the Kenyan NAP for 2015–2030 specified mainstreaming climate change adaptation into CIDPs as a priority action [11].

### Knowledge gap and research objectives

We are not aware of existing frameworks for evaluating the extent to which planning activities address climate change adaptation as a health sector issue or health as an environmental or climate issue. This research aims to develop and apply a framework for evaluating the extent to which subnational plans address specific actions and interventions related to health and climate change as put forth in national frameworks. Beyond the evaluation of this assessment framework, this research aims to identify Kenyan counties that are considering climate change and health in their planning and those that may need additional support to address this challenge.

## Methods

### Evaluating CIDPs

Given the lack of an existing framework for governments to evaluate climate change adaptation planning for the health sector, international frameworks and Kenya-specific policies listed in Table 1 were examined to develop such a framework to be used in assessing county planning through the examination of CIDPs.

**Table 1:** Key frameworks and policies regarding adaptation to climate change.


UNFCCC FRAMEWORK

• National Adaptation Plans

**WHO FRAMEWORKS**

• Health in National Adaptation Plans

• Quality Health National Adaptation Plans

• Framework for Public Health Adaptation to Climate Change

**AFRICAN FRAMEWORK**

• African Framework for Public Health Adaptation to Climate Change

**KENYAN POLICIES**

• Kenya National Adaptation Plan: 2015–2030

• Kenya National Climate Change Response Strategy (KNCCRS)

• Climate Change Act 2016


CIDPs have four main sections (County General Information, Links to Other Plans, Review of Previous CIDPs, and County Development Priorities and Strategies) within which sub-sections address sectors such as health, agriculture, tourism, and the environment. The four sections of the CIDP were evaluated regarding the degree to which the joint consideration of climate change and health is present. Table 2 lists the evaluation elements developed for evaluating the CIDPs. The joint consideration of climate change and health was evaluated in multiple ways within each section of the CIDPs.

**Table 2:** CIDP evaluation elements.


Section 1: County description	Was climate change mentioned in the environmental sector? Was health mentioned in the context of climate change within the environmental sector? If so, how many specific climate-sensitive health conditions were noted?

Section 2: Links to other plans	Was Sustainable Development Goal 13 mentioned? Was Kenya Vision 2030 Medium Term Plan III Climate Change Goal mentioned?

Section 3: Review of previous CIDPs	Did the previous 5-year CIDP note adaptation for climate change in the health sector?

Section 4: Priorities and strategies—health sector	Was building adaptive capacity for climate change mentioned in the health sector? Is a climate change adaptive capacity program planned? If so, is it a full- or sub-program? If any key program outputs are noted, what are they?

Section 4: Priorities and strategies—environment sector	Was building adaptive capacity for climate change or mitigating climate change mentioned in the environment sector? To what extent is climate change prioritized? Is a climate change adaptive capacity program planned? If so, is it a full- or sub-program? If any key program outputs are noted, what are they?


Data from each section of each CIDP were abstracted into a spreadsheet. Once this was completed for all 47 counties, descriptive statistics were run to summarize the extent to which counties jointly considered climate change and health in their integrated development plans. In addition to summarizing this data, illustrative quotes from a subset of CIDPs were pulled to complement the presence/absence data.

### Were ACCAPAHS interventions utilized in CIDP adaptation strategies?

Health sector programs planned for climate change adaptation were evaluated based on the extent to which they addressed the ACCAPAHS interventions. Table 3 lists the ACCAPAHS interventions and the metrics used to assess planned programs noted in each CIDP.

**Table 3:** County-level interventions specified by ACCAPAHS measured in Kenyan CIDPs and how they were measured.


INTERVENTIONS	EVALUATION METRIC: DOES THE CIDP ADDRESS THE FOLLOWING?

1. Undertake baseline risk and capacity assessments	The need to undertake these assessments

2. Capacity building	Increasing the number of healthcare workers, increasing hospital beds, strengthening healthcare infrastructure

3. Implement integrated environment and health surveillance	Action to increase data sharing/health surveillance

4. Undertake awareness raising and social mobilization	Specific action to increase awareness of climate-sensitive diseases among the public (such as communicable, vector, etc.)

5. Promote public-health oriented environmental management	Program or sub-program on health promotion

6. Scale up existing public health interventions	Scale up existing public health actions focused on environmental factors WASH, communicable and vector-borne diseases

7. Strengthen and operationalize the health components of disaster risk reduction	Disaster preparedness in the health sector development priorities or cross-sectoral collaborations

8. Promote research on climate change impacts	Allocating funds for research in the health sector

9. Strengthen partnerships and intersectoral collaboration	Cross-sectoral impacts relating to adaptation in the health sector


### Composite score of CIDP and ACCAPAHS evaluation

After the above assessments of CIDPs were complete, a climate and health adaptation (CHA) score for each county was calculated. CIDP and ACCAPAHS elements were given a score of 1 if present and 0 if absent except for a few CIDP elements. In section 4 of the CIDPs, counties were given a score of 0 if adaptive capacity was not mentioned, a score of 1 if adaptive capacity was mentioned but no programs addressed it, a score of 2 if there is an adaptive capacity sub-program and a score of 3 if there is a full program. The sum of scores from all evaluation elements was calculated. The lowest possible score of 0 and the highest possible score of 23. Based on the distribution of the data, scores were assigned to three categories, low (≤5), medium (5 < x < 11) high (≥11) joint consideration of climate change and health in CIDPs. Each county was assigned to one of these groups based on their CHA score. The data on CHA scores was then applied to Kenyan shape files in ArcGIS to examine the geographic distribution of levels of joint consideration of climate change and health.

### Associations between county poverty rates and CHA scores

To evaluate whether CHA scores are related to poverty rates, county-level poverty rate data was obtained from the Kenya poverty report for 2021 by the Kenya National Bureau of Statistics [26].Because CHA scores and poverty rates were normally distributed, Pearson correlation analyses were conducted.

All statistical analyses were conducted using SAS version 9.4 (SAS Institute, Cary, NC).

## Results

### County-level CIDPs: Climate change and health in “County Description” and “Links to Other Plans” sections

As seen in Table 4, even though almost all counties in Kenya mention climate change in the county description, only half mention health in the context of climate change. Likewise, nearly all counties link their development plan to sustainable development goal 13 but only a third link to the climate change goal in Kenya Vision 2030 MTP III. None of the CIDPs mentioned climate change in the context of health in previous CIDPs. Although climate change is noted in CIDPs of nearly all counties, the consideration of health in the climate change/environment section is far less common.

**Table 4:** Summary of findings regarding the joint consideration of climate change and health in the first two sections of CIDPs.


MEASURE	YES	NO

Climate change is mentioned in the county description	45 (95.7%)	2 (4.3%)

Health is mentioned in the climate change/environment county description	23 (48.9%)	24 (51.1%)

Linked to Sustainable Development Goal 13	43 (91.5%)	4 (8.5%)

Linked to Kenya Vision 2030 Medium Term Plan III—Climate change goal	16 (34.0%)	31 (66.0%)


### Analysis of the “Priorities and Strategies” section

Table 5 summarizes key outputs, sub-programs, and full programs noted in the “Priorities and Strategies” section of CIDPs. These results further demonstrate the stark contrast among counties based on their joint consideration of climate change and health. Over 50% of counties have a sub or full program for building adaptive capacity to climate change, whereas there are no full programs on environmental health and only 45% of counties have a sub-program addressing environmental health. Additionally, only 12 of the 47 counties have both an environmental health and adaptive capacity sub-program.

**Table 5:** Summary of the priority given the climate change and health in two development priority sections of CIDPs.


	ENVIRONMENTAL HEALTH IN HEALTH SECTOR DEVELOPMENT PRIORITIES				

		*SUB-PROGRAM*	*MENTIONED*	*NOT MENTIONED*	*TOTAL N (%)*

Climate change adaptive capacity or mitigation goal in the development priorities	*Full program*	1	0	4	5 (10.6%)

	***Sub-program***	12	2	8	22 (46.8%)

	***Mentioned***	5	1	3	9 (19.14%)

	***Not mentioned***	3	0	8	11 (23.4%)

	***Total N (%)***	21 (44.7%)	3 (6.4%)	23 (48.9%)	47 (100%)


The health sector was evaluated for the number of key outputs specified that would build adaptive capacity to climate change, such as having a backup generator. As seen in Table 6, there is a strong association between the health sector mentioning adaptation strategies as key outcomes and mentioning one or more specific climate-sensitive health impacts. Compared to county CIDPs that did not note health sector adaptation strategies as key outcomes, those that did appear to be more likely to also mention health impacts (odds ratio 3.11, 95% confidence interval 0.60–16.02).

**Table 6:** CIDPS with health sector adaptation goals by mentioning specific climate-sensitive health outcomes in the background.


	PRIORITIES AND STRATEGIES FOR THE HEALTH SECTOR—ADAPTATION STRATEGIES AS KEY OUTCOMES			

		*NOT MENTIONED*	*MENTIONED*	*TOTAL*

Climate sensitive health impacts in the background	*Not mentioned*	28	2	31 (66%)

	*Mentioned*	12	4	16 (34%)

	*Total*	40 (85.1%)	7 (14.9%)	47 (100%)


### Analysis of ACCAPAHS-specific actions

Following the initial evaluation of all 47 CIDPs, we further analyzed the 24 counties that listed an environmental health subprogram or adaptation strategies in the health sector based on the specific ACCAPAHS actions that were addressed. As seen in Table 7, these 24 counties prioritized capacity building, environment, and health surveillance, and scaling up existing public health interventions but are lacking in baseline risk and capacity assessments. Few counties addressed efforts to raise awareness or to mobilize the population of the county about climate change and health (7), to promote health components of disaster risk reduction (5), or to conduct research on climate change impacts (6).

**Table 7:** Evaluation of ACCAPAHS action presence in the health sector development priorities for 24 Kenyan counties.


ACCAPAHS ACTION	NUMBER (%) OF COUNTIES WITH THIS ACTION

Undertake baseline risk and capacity assessments	0 (0)

Capacity building	24 (100)

Implement integrated environment and health surveillance	21 (87.5%)

Undertake awareness raising and social mobilization	7 (29.2%)

Promote public-health-oriented environmental management	14 (58%)

Scale up existing public health interventions	24 (100)

Strengthen and operationalize the health components of disaster risk reduction	5 (20.1%)

Promote research on climate change impacts	6 (25%)

Strengthen partnerships and intersectoral collaboration	12 (50)


### Composite scores of county-level planning for climate change and health

**Figure 1:**
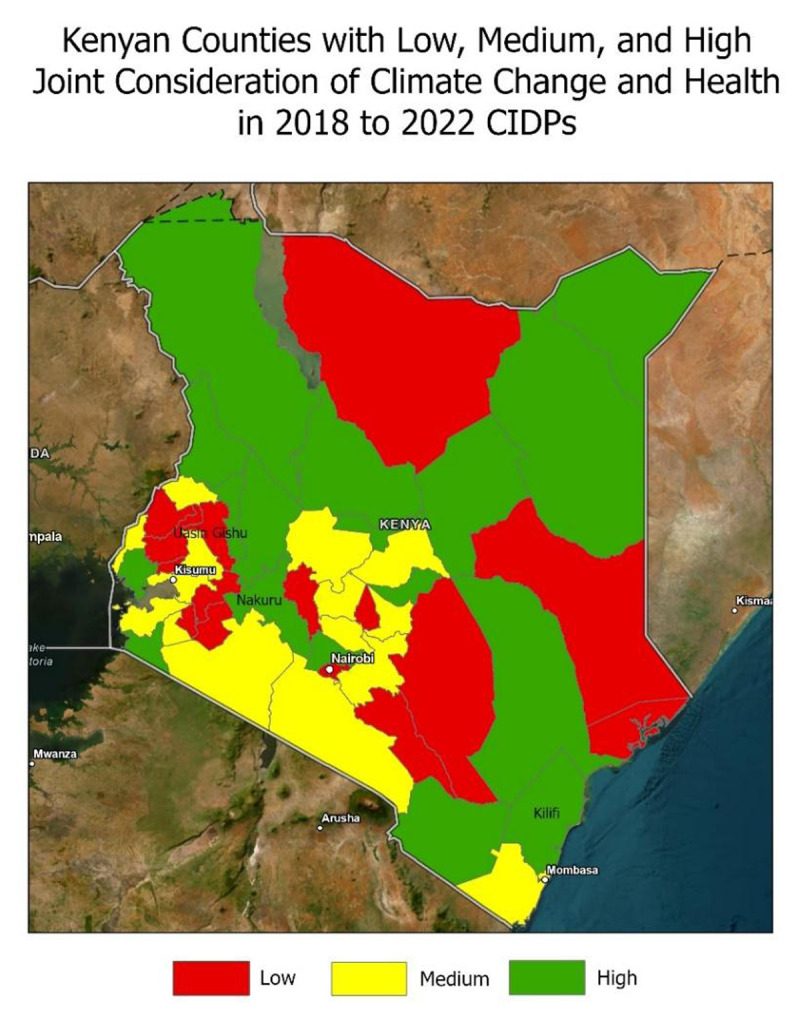
Map of Kenyan counties by degree of connection between climate change and health in 2018–2022 CIDP.

After evaluation of CIDPs based on the CIDP framework and the ACCAPAHS framework, a composite score was calculated with scores ranging from 1 to 15 (higher scores indicate greater attention to climate change impacts and adaptation in the health sector in CIDPs) out of a possible score of 23, with a median score of 8. Based on the distribution of the scores, counties were classified into low, medium, and high composite score groups. As seen in Figure 1, composite scores vary drastically across the country and do not follow a gradient or regional pattern. Kilifi and Nakuru counties have the highest composite score of 15, and Uasin Gishu county has the lowest composite score of 1. The poverty rate ranged from 16.5% to 77.7% among the counties. Poverty rates were not significantly correlated with CHA scores (Pearson correlation coefficient of 0.255, *P* = 0.08).

## Discussion

While nearly all counties in Kenya developed CIDPs that note climate change in the context of development, only half mention health in the context of climate change in the CIDP “County Description” section. Sixteen of the counties (34%) noted one or more specific climate-sensitive health outcomes in their discussions of the health impacts of climate change. In the “Development Priorities” section, 12 (25.3%) counties had a sub-program for both adaptive capacity to climate change and environmental health. Further, 24 (51%) counties prioritized an environmental health subprogram and/or adaptation strategies in the health sector. While all 24 of these counties specified capacity building and scaling up public health interventions in the health sector, none specified conducting baseline risk and capacity assessments, <30% specified increasing research on climate change, integrating health into disaster risk reduction, and raising awareness. CHA scores show no clear spatial pattern and were not correlated with county level poverty rates. This suggests that county-level poverty does not drive the extent of climate change preparedness and that health departments of counties with low CHA scores should be prioritized for education, training, and support.

Variability in subnational adaptive capacity has been seen in previous subnational vulnerability assessments, but unlike our results, they followed a south-to-north gradient [27]. The measure of adaptive capacity by S.N. Marigi, was a function of literacy rates and poor health services and as a result was highly correlated with the SES of the counties [27]. Given that our CHA score did not include measures of SES and S.N. Marigi’s adaptive capacity score did not include policy measures, the disconnect between findings is not surprising [27]. Understanding the extent to which adaptive capacity is being addressed in subnational planning is essential to understanding county-level planning needs and to guide resource allocation. Rather than requiring county administrators to take on new tracking requirements for evaluating the extent of climate and health adaptation at the county level, the assessment of existing planning documents may be useful while not increasing reporting requirements. Kenya’s CIDPs provide some insight into the extent that subnational planning documents can be used to evaluate the preparedness of the health sector for climate change. The use of existing planning processes to prepare for climate change is consistent with the 2015–2030 Kenyan National Adaptation Plan which promoted the mainstreaming climate change adaptation into CIDPs [11]. Additionally, strengthening the integration of climate change adaptation into the health sector was specified, but this intervention has a miniscule budget compared to the other sector-specific interventions, with a budget of 40 million USD compared to 20 billion USD in the infrastructure sector [11].

If health planning and climate change adaptation planning are done in concert, the results would, potentially, be better than if they were considered separately. Climate change alters how and where population health is impacted by factors such as flooding, drought, temperature, and the distribution of vector-borne and zoonotic diseases. Additionally, healthcare facilities may require additional resources to respond to a larger number of cases of diarrheal disease. By considering climate change in planning the future needs of local healthcare systems and healthcare facilities, the result should be better preparedness, increase international funding, reduced future vulnerability, and smaller gaps between climate change risk and preparedness [6,14].

This evaluation framework of Kenyan CIDPs and our conclusions about the readiness of county planners for the health impacts of climate change have several limitations. First and foremost, this evaluation framework has not yet been validated against observed differences in the burden of climate-sensitive disease at the county level. We analyzed county-level development plans to develop CHA scores; it is unknown whether CHA scores reflect metrics of health system adaptive capacity such as the number of hospital beds, resilience of health care facility structures and infrastructure, vector control programs, or climate hazard response capabilities in these counties. Second, it is likely that different data sources and the use of weighting factors to calculate composite scores may be more predictive of the extent to which county planning is preparing for the local impacts of climate change on health systems. Third, it is not known to what extent this approach would be transferrable to other LMICs. To address this limitation, this evaluation framework would need to be applied to other subnational development plans in other LMICs. Fourth, this framework for evaluating plans for health adaptation at the subnational level is based on specific actions and interventions laid out in frameworks—NAP, HNAP, and ACCAPAHS—that have been developed for use at the national level. Given this change in spatial scale, the evaluation metrics may not accurately capture the true joint consideration of climate change and health on a subnational scale. Specifically, county-level governments may not have the resources to increase adaptive capacity for health even if they did address them in the CIDPs. Fifth, the measures used to evaluate the CIDPs were proxies for the actions mentioned in the NAP, HNAP, and ACCAPAHS and were based on the information present in Kenyan CIDPs. Therefore, the results are not a precise evaluation of the extent that the specific actions were implemented. This study did not evaluate greenhouse gas emissions of the health sector or approaches to mitigating those contributions to climate change. Globally, the healthcare sector contributes 4.4% of the net emissions of greenhouse gases [28]. In the KNCCRS and other Kenyan policies or reports, the only mitigation measures mentioned by the health sector is adding green space and increasing the promotion of using low-carbon methods of transportation among patients. Since this evaluation framework is based on adaptation plans that do not address how the health sector can contribute to mitigation, mitigating climate change is not represented in this analysis.

To address the limitations mentioned above, future research could address the extent to which these estimates are predictive of health sector adaptive capacity at the subnational scale. For that to occur, valid metrics of adaptive capacity that make use of readily available data are needed. This can be done by utilizing the composite climate and health adaptation (CHA) scores as a predictor of other metrics of adaptive capacity in counties in Kenya. Secondly, further exploration of what county-level factors could be driving the difference in CHA scores, are there differences in climate hazards, sensitivity, or other structural factors playing a role. Third, the extent to which this evaluation framework transfer from CIDPs in Kenya to different subnational plans in another sub-Saharan African country needs to be evaluated. Specifically, reapplying this framework to the next round of CIDPs in Kenyan or county-level development plans in another sub-Saharan African country. Despite limitations, it is apparent that there is a wide range of the extent to which county planners address the adaptive capacity of counties in Kenya regarding the health impacts of climate change, with some counties lagging far behind others. Therefore, resources to support planning by county governments for increasing health sector adaptive are needed, as are resources to implement those plans. As an initial step, additional support for the counties with low CHA scores should be expedited.
